# An Integrated Pipeline for de Novo Assembly of Microbial Genomes

**DOI:** 10.1371/journal.pone.0042304

**Published:** 2012-09-13

**Authors:** Andrew Tritt, Jonathan A. Eisen, Marc T. Facciotti, Aaron E. Darling

**Affiliations:** 1 Genome Center, University of California Davis, Davis, California, United States of America; 2 Department of Evolution and Ecology, University of California Davis, Davis, California, United States of America; 3 Department of Medical Microbiology and Immunology, University of California Davis, Davis, California, United States of America; 4 Department of Biomedical Engineering, University of California Davis, Davis, California, United States of America; Wayne State University, United States of America

## Abstract

Remarkable advances in DNA sequencing technology have created a need for *de novo* genome assembly methods tailored to work with the new sequencing data types. Many such methods have been published in recent years, but assembling raw sequence data to obtain a draft genome has remained a complex, multi-step process, involving several stages of sequence data cleaning, error correction, assembly, and quality control. Successful application of these steps usually requires intimate knowledge of a diverse set of algorithms and software. We present an assembly pipeline called A5 (**A**ndrew **A**nd **A**aron's **A**wesome **A**ssembly pipeline) that simplifies the entire genome assembly process by automating these stages, by integrating several previously published algorithms with new algorithms for quality control and automated assembly parameter selection. We demonstrate that A5 can produce assemblies of quality comparable to a leading assembly algorithm, SOAPdenovo, without any prior knowledge of the particular genome being assembled and without the extensive parameter tuning required by the other assembly algorithm. In particular, the assemblies produced by A5 exhibit 50% or more reduction in broken protein coding sequences relative to SOAPdenovo assemblies. The A5 pipeline can also assemble Illumina sequence data from libraries constructed by the Nextera (transposon-catalyzed) protocol, which have markedly different characteristics to mechanically sheared libraries. Finally, A5 has modest compute requirements, and can assemble a typical bacterial genome on current desktop or laptop computer hardware in under two hours, depending on depth of coverage.

## Introduction

High throughput DNA sequencing continues to revolutionize our understanding of biological systems. In particular, the *de novo* sequencing and assembly of genomes and metagenomes has yielded basic scientific insight into the relationship between genotype and phenotype, in addition to biotechnological advances in enzyme discovery, bioprospecting, medicine, and agriculture. Although many high throughput sequencing instruments have been developed, such as the ABI SOLiD, Helicos Heliscope, IonTorrent PGM, Roche 454, and Pacific Biosciences RS1, we focus on data generated by the Illumina instrument in this work because it is the most prevalent data type at the moment. Current Illumina instruments such as the HiSeq 2000 and MiSeq generate paired nucleotide sequence reads of length up to 150 nt per read from fragments as long as 600 nt, with longer reads and inserts under development. Currently one run of a HiSeq 2000 instrument generates up to 600 Gbp of sequence data. Despite the widespread use of Illumina sequencing, *de novo* genome assembly from Illumina data continues to pose a challenging problem.

A tremendous number of software tools have been developed to assist with genome assembly from Illumina data. These include tools for base calling of the images from the sequencer [1], [2], error correction of the sequence reads [3], [4], removal of adapter sequence contamination [5], contig assembly [6], [7], and scaffolding [8]–[10]. This list is not complete, but rather meant to illustrate some of the many tools for Illumina sequence analysis. As part of a project to sequence and assemble *de novo* the genomes of 64 halophilic archaea, we have evaluated many of these software tools and constructed a new genome assembly pipeline that incorporates methods for data cleaning, error correction, contig assembly, and scaffolding together with a new algorithm for assembly quality control.

The new assembly quality control algorithm uses paired-end read information to detect and fix misassembled contigs and scaffolds. The first stage involves mapping reads back to assembled contigs. The second stage involves detecting statistically significant clusters of read pairs that conflict with the assembled contigs. Having identified putative regions of misassembly, the algorithm then determines the region of misassembly as precisely as possible and removes that region from the assembly, breaking the contigs or scaffolds at that point. The quality control algorithm is implemented in a software module that can also be used independently of the assembly pipeline.

The new assembly pipeline, called A5, can operate directly on FastQ format data generated by an Illumina sequencing run without any prior processing. The A5 pipeline also contains methods to infer many of the assembly parameters directly from the data, and in cases where that was not practical, default values have been chosen by tuning their values on assemblies of *Haloferax mediterranei* and *Haloferax volcanii* DS2 [11], for which high quality reference genomes were available.

We present comparison of the A5 pipeline's performance relative to the SOAPdenovo assembler [12] on two datasets wherein we attempted to fix the *human time budget*, in terms of the number of steps that must be performed by a user, to be roughly equal for the two approaches. We also compare against SOAPdenovo in an ideal setting where extra effort has been taken to clean and error correct the reads prior to use of SOAPdenovo. The first dataset is the halophilic archaeon, *H. volcanii* DS2, for which a high quality published reference genome is available [11]. For this dataset we are able to use reference-based assembly metrics to evaluate assembly quality [13]. We also evaluate assembly quality on an *Escherichia coli* CC118 isolate sequenced using transposon-catalyzed library preparation methods (Epicentre Nextera). No high quality reference genome exists for the *E. coli* isolate, so we report basic descriptive statistics for assemblies generated by each method.

*De novo* genome assembly from Illumina data is an extremely active area of research, with many assembly algorithms published and many more continuing to be produced. A thorough comparison of the performance of all these methods is a highly nontrivial undertaking and well outside the scope of the present work. Instead, we chose to compare A5 to a single other widely-used assembly method, namely SOAPdenovo. We selected SOAPdenovo for comparison because it ranked among the best in two recent surveys of assembly algorithms [14], [15], because it is able to run on a single paired-end library, and like A5 is relatively simple to download, install, and use. Although methods that require both small insert paired-end libraries and large insert mate-pair libraries can produce very high quality results [16], the time, cost and technical expertise required to construct large insert libraries is significantly beyond that required for small insert libraries (especially using transposon-catalyzed library construction). For this reason we feel there is a great need for methods to easily produce assemblies of the highest quality possible without large insert mate-pair data. A5 can be considered a first attempt at such a method.

Although the A5 pipeline was parameterized using archaeal genomes, it is readily applicable to genome assembly of other organisms including bacteria, virii, and homozygous eukaryotes. Compute requirements are likely to be the limiting factor for assembly of large genomes; these requirements are discussed below.

## Results

We evaluated the performance of A5 on two real Illumina data sets and compared the results to those obtained when running SOAPdenovo v1.05 [12] on the same datasets. The first data set (called **Volc**) is a paired-end short insert library constructed from *H. volcanii* DS2 genomic DNA using sonication followed by end-repair, A-tailing, and adapter ligation, and was sequenced on an Illumina GAIIx instrument. Sequencing yielded 6844701 read pairs, with each read being 78 nt in length. These data have been deposited at the NCBI Short Read Archive, accession SRX105348 (data can be downloaded from http://edhar.genomecenter.ucdavis.edu/ 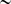
andrew/ngopt_pipeline/ms/). We chose *H. volcanii* for this evaluation because it is a model organism among the archaea, we have an ongoing project to sequence 64 other haloarchaea genomes, and a high quality reference genome is available for *H. volcanii* DS2 [11], enabling the use of reference-based assembly metrics [13]. The second data set, called **Tn** and previously published by [17], is a paired-end library constructed from *E. coli* CC118 genomic DNA using transposon-catalyzed adapter ligation (Nextera) and was sequenced on an Illumina HiSeq 2000 instrument using TruSeq 2 chemistry. Reads from this dataset were obtained from the NCBI Short Read Archive, accession SRX030179.

We executed A5 and SOAPdenovo for each data set. Table 1 reports the assembly performance for **Volc** assemblies. Table 2 reports the assembly performance for **Tn** assemblies. **Volc** assemblies were scored using Mauve Assembly Metrics [13], which quantifies differences between the reference and assembly using whole genome alignment. We note that aligner error may cause additional errors to be found between the assembly and the reference. Although high quality reference assemblies exist for other *E. coli* isolates, none are available for strain CC118. We can not use another *E. coli* as a reference due to the potential for extensive genomic divergence among *E. coli* isolates [18]. Contigs from A5 were broken using the A5QC algorithm. **Volc** contigs were broken up into 859 contigs (N50 = 8170) and **Tn** contigs were broken up into 342 contigs (N50 = 27316). N50 is defined as the contig length N for which 50% of all bases in the assembly are in a contig (or scaffold) of length 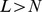.

**Table 1:** Assembly metrics for *H. volcanii* DS2.

	SOAPdenovo						A5		
Assembly	ctg-CDS	scaf-CDS	ctg-N50	scaf-N50	ctg-LCB	scaf-LCB	ctg	scaf	scaf-QC
Sequence count	7686	211	10258	212	19508	226	853	106	95
N50	5111	125642	4775	125739	2832	107081	8170	101041	110196
Miscalled bases	379	573	270	395	409	377	120	315	247
Uncalled bases	0	92290	0	11430	0	13304	0	6436	6727
Extra bases	269960	18582	27069	14295	32797	19732	14096	17903	18496
Missing bases	149253	151966	148291	142017	160773	156254	128909	107421	106626
Extra sequences	6129	75	8604	76	17194	95	33	5	5
Missing replicons	0	0	0	0	1	1	0	0	0
DCJ Distance	1559	143	1656	140	2317	134	839	123	100
LCB Count	15	22	9	14	7	10	45	55	28
Broken CDS	434	434	454	454	634	634	276	214	212

Reference-based assembly metrics on ten assemblies of *H. volcanii* DS2 (**Volc**) dataset. “scaf” indicates an assembly that has been scaffolded, while “ctg” indicates no scaffolding. Labels “-CDS”, “-N50”, and “-LCB” indicate SOAPdenovo assemblies run with parameter combinations that minimized broken coding sequences, maximized scaffold N50, and minimized LCB (Locally Collinear Block) count, respectively. For A5, assembly “scaf-QC” has been broken using the A5QC algorithm and rescaffolded using SSPACE. The DCJ Distance is the Double-Cut-and-Join distance [35], a measure of the minimum number of rearrangement operations required to transform one genome assembly into another.

**Table 2:** Assembly metrics for *E. coli* CC118.

	SOAPdenovo		A5		
Assembly	ctg	scaf	ctg	scaf	scaf-QC
Sequence count	4348	197	323	111	87
N50	12825	83067	27846	72166	82719
Mean seq len	1056	22647	13800	40207	51300
Max seq len	48725	200327	113049	330689	330689
Total bases	4590705	4461465	4457409	4462953	4463084
Uncalled bases	0	26290	0	220	290

Non-reference based metrics on assemblies of *E. coli* CC118 (**Tn**) dataset. Data for SOAPdenovo were calculated from the assembly run with parameters that maximized scaffold N50. “scaf” indicates an assembly that has been scaffolded, while “ctg” indicates no scaffolding. For A5, assembly “scaf-QC” has been broken using the A5QC algorithm and rescaffolded using SSPACE.

We initially ran SOAPdenovo with default parameters; however, the resulting assemblies were of extremely poor quality. For **Volc** there were 280985 contigs (N50 = 76) and 14433 scaffolds (N50 = 209), and for **Tn**, there were 1572720 contigs (N50 = 76) and 8144 scaffolds (N50 = 121). Rather than reporting poor results for SOAPdenovo, we endeavored to manually optimize its assembly parameters so that we can compare the A5 assembly to the best possible SOAPdenovo results. To do so, we ran SOAPdenovo with different combinations of values for the parameters 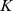 and 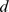, where 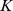 is the 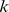-mer size for SOAPdenovo and 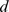 is the threshold for the minimum number of times a 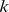-mer must be observed in the data to be considered valid. For both datasets, we selected combinations that maximized scaffold N50. In addition, for the **Volc** dataset we also present assembly scoring results for the parameter combination that minimized LCB (locally colinear block) count between the assembly and the reference, as well the combination that minimized the number of broken coding sequences. The parameter combination that maximized scaffold N50 for **Volc** also minimized the sum of missing and extra bases relative to the reference. The parameter space queried was 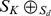, where 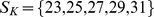 is the set of values over which 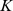 was varied, and 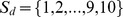 is the set of values over which 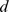 was varied. Using this process, the optimal parameters were found to be

- **Volc** (-N50): 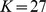, 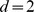.
- **Volc** (-LCB): 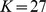, 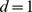.
- **Volc** (-CDS): 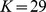, 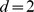.
- **Tn**: 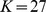, 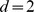

Labels “-CDS”, “-N50”, and “-LCB” indicate SOAPdenovo assemblies run with parameter combinations that minimized broken coding sequences, maximized scaffold N50, and minimized LCB (Locally Collinear Block) count, respectively. Locally collinear blocks are continuous regions of assembly which can span zero or more contigs and scaffolds which are free from rearrangement relative to the reference genome. See [13], [19] for a more complete discussion. A5 assemblies were generated using source code from revision 625 of A5.

**Figure 1:**
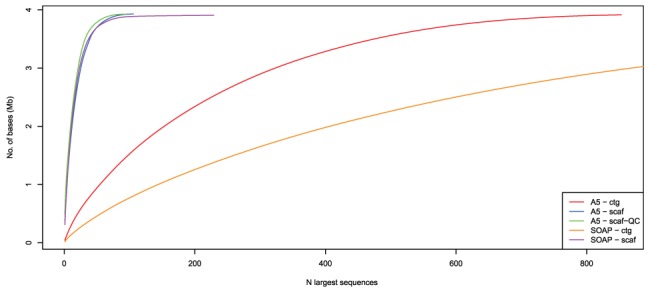
Sequence length accumulation curve for six assemblies of the model archaeon *H. volcanii* DS2. Curves represent the number of bases in an assembly as a function of the 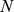 largest sequences. Assemblies generated from SOAPdenovo and A5 are labelled with “SOAP” and “A5”, respectively. “scaf” indicates an assembly that has been scaffolded, while “ctg” indicates no scaffolding. For A5, assembly “scaf-QC” has been broken using the A5QC algorithm and rescaffolded using SSPACE. A perfect assembly would have exactly the number of sequences as the organism has replicons (5 in this case), and the curve would be in the extreme upper left corner.

In addition to the reference-based assembly metrics, we present the scaffold and contig size distribution as a “length accumulation curve” in Figure 1. In that figure, scaffolds (contigs) are sorted in descending order by length and the cumulative length is plotted as additional scaffolds (contigs) are added. The scaffold length distribution for A5 and SOAPdenovo appear to be very similar. However, the contig length distributions are quite different, with SOAPdenovo generating much shorter contigs than A5. This exemplifies a fundamental difference between the contig-generation strategies employed by SOAPdenovo and IDBA (used in the A5 pipeline). SOAPdenovo is conservative during contig generation and avoids introducing misassembly and chimerism, but produces only very short contigs, whereas IDBA produces long contigs that occasionally contain misassemblies that the A5 pipeline's QC step must resolve.

### A5 and SOAPdenovo assemblies on error corrected reads

As reported elsewhere [15], assemblers such as SOAPdenovo can be highly sensitive to errors in the read sequence data and cleaning and filtering the reads prior to assembly can offer large improvements in some cases. In the previous section we report results of a comparison between A5 and SOAPdenovo assemblies when each pipeline is run in a single step from raw Illumina data. However, common practice involves manually performing several read cleaning steps prior to a SOAPdenovo assembly.

A direct comparison of A5 to SOAPdenovo is challenging because A5 incorporates read cleaning steps whereas SOAP does not. Therefore, we also ran SOAPdenovo assemblies of **Volc** and **Tn** on cleaned and error corrected reads generated by stage 1 of the A5 pipeline. For these assemblies we also scanned a larger range of possible 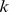-mer sizes for SOAPdenovo at the suggestion of an anonymous reviewer: 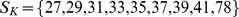. The -CDS, -N50, and -LCB metrics were optimal in scaffold assemblies with 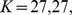 and 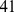, respectively.

SOAPdenovo assembly results on cleaned reads are provided in Table S2. As expected, using cleaned reads reduced the number of miscalled bases by 37–59% in the SOAPdenovo contig assemblies. Surprisingly, the number of miscalled bases in scaffold assemblies was lower only for 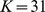, with scaffold assemblies at other 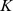 values having higher miscalled base counts than the optimal -CDS, -N50, and -LCB runs on uncleaned data. We speculate that this may be result from two factors. First, the currently available version of SOAPdenovo does not support setting 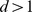 when 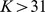. Therefore all assemblies on clean data with higher 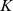 settings were done with with 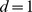. However we observed that 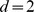 produced better assemblies when 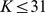 in some settings (see above). Second, the scaffold gap filling process used by SOAPdenovo may explain the extra error in scaffold assemblies relative to contig assemblies. Scaffold gap filling identifies reads with pairing information suggesting they belong in the scaffolded region between two contigs and adds them to the assembly. In some cases these regions might have low coverage, making error correction less effective.

In comparing the SOAPdenovo results on raw and cleaned reads, we observe that the highest achieved scaffold N50 and the LCB count metric (a measure of misassembly error) are apparently unaffected by the read cleaning process, with a difference of only 0.1% between cleaned and raw data in scaffold N50, and no change in LCB count. We observe a slight reduction in contig N50 and increase in broken CDS when SOAPdenovo is run on cleaned reads.

### Compute time and memory requirements

The A5 pipeline can construct genome assemblies with limited memory and CPU requirements. For microbial genomes around 4 Mbp sequenced to 100× coverage, memory as low as 4 GB can be sufficient (data not shown). Typically the error correction using SGA is the most resource intensive stage in the pipeline. SGA's implementation of error correction offers a configurable space/time tradeoff wherein temporary files on a filesystem can be used to reduce RAM requirements at the expense of extra compute time. A5 makes use of this configurable tradeoff by determining at runtime the available memory on the system and allocating a fixed fraction of it to SGA for error correction. This approach enables error correction to run faster on machines with larger available memory.

The A5 assemblies of **Volc** and **Tn** were conducted on a 8 core 64-bit system with 48 GB RAM. On this machine, the **Volc** assembly took 1 hour, 20 minutes with a peak memory usage of 20 GB during the SGA error correction step. The **Tn** assembly completed in 1 hour, 29 minutes with a peak memory usage of 21 GB.

## Discussion

When constructing assemblies directly from Illumina sequence output in a single step, A5 produces higher quality assemblies than SOAPdenovo on the datasets we analyzed. In particular, A5 assemblies have a 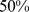 lower rate of broken coding sequences relative to SOAPdenovo assemblies. For gene-oriented analyses such as inferring metabolic potential and surveys of natural selection via dN/dS ratios, the reduced error in coding sequences may be very advantageous. To obtain the SOAPdenovo results we conducted a parameter sweep over 50 combinations of 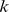-mer length (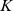) and the minimum 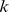-mer frequency (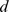), while A5 required only a single run of the pipeline. SOAPdenovo outperforms A5 in scaffold N50 on the *H. volcanii* DS2 dataset, but on the *E. coli* dataset (for which no high quality reference assembly is available) A5 produces a better assembly when measured by scaffold count, mean scaffold size, and max scaffold size. The scaffold N50 of SOAPdenovo on the *E. coli* was higher by about 1%. One possible reason that A5 may produce better results on the transposon catalyzed library is that the insert size using that library preparation protocol often does not fit a normal distribution. Instead the insert size distribution depends greatly on the relative concentrations of transposase and target DNA and can range from a truncated uniform to roughly lognormal depending on the enzyme concentration and what size selection steps are taken during library preparation. Most scaffolding programs to-date model the insert sizes for paired-end reads using a normal distribution with a particular mean and standard deviation. A5 also uses this model, but has been configured to be permissive of scaffolding using libraries with broadly distributed insert sizes. We speculate that another explanation for A5's improved performance on transposon-catalyzed libraries may be that the method is more robust to low coverage regions. Illumina libraries constructed by in-vitro transposition with Tn5 transposase have considerable target site preference (data not shown), leading to highly nonuniform coverage around a genome.

In all cases where reference data was available A5 produced fewer miscalled bases. This is to be expected, as A5 first performs error correction on reads before assembling them into contigs. Running SOAPdenovo on error corrected reads did reduce base call errors in assembled contigs, however both contig and scaffold assemblies still had more basecall errors than A5's assemblies. A5 also produced assemblies with 50% fewer broken CDS than SOAPdenovo when run on our data. This may have important implications for downstream analysis of gene function, regulation, and metabolism.

The strategy used for detection of misassmblies demonstrates the utility of paired-end data for improving draft genome assemblies. In addition to identifying misassemblies after scaffolding, paired-reads may also be used to identify repetitive regions. Although we use paired short reads, the methodology is not limited to this type of data. Long reads with split mapping positions could in theory be used in the same manner as the paired short read data.

### Limitations and scope

A limitation to misassembly detection is the underlying assumptions about the structure of misassemblies. The first assumption we make is that the only feature of the misassembly is a false adjacency between two bases. In many cases, however, a misassembly consists of more than a single false adjacency and includes extra inserted sequence. One approach to overcome this would be to employ a model that characterizes the insertion of additional sequence. A related limiting assumption is that coverage within each of the two regions surrounding the misassembly is uniform. This assumption is frequently violated, as sequence coverage is rarely uniform. We also assume that coverage is equal on both sides of the false adjacency. In cases where coverage is not equal between the two regions flanking a misassembly, as may be the case in metagenomes, a spatial clustering algorithm that allows for variable density clusters, such as AMSTLSC [20], would more accurately identify blocks. Finally, we assume that all replicons in the target genome are circular. In genomes containing linear chromosomes, a misassembly combining a whole linear chromosome with a position internal to another chromosome would result in a single block on one side of the misassembly. Identifying a misassembly in this case would require additional information. When two chromosomes have been assembled together at their ends (telomeres, for chromosomes with such structures), no such blocks will be found necessitating a different approach to identifying misassemblies.

In addition to theoretical limitations, A5 also also has practical computational limits. Large datasets, such as a full lane of data generated on the Illumina HiSeq 2000 platform, require resources beyond that typically available in a desktop or laptop computer. The major computational bottlenecks of A5 are the first two stages: read cleaning and contigging. Memory requirements for read error correction grow with total data volume, requirements for contigging grow with data volume and total size/complexity of the assembled genome (since the *de Bruijn* graph is more complex in these cases). The DBSCAN algorithm has 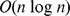 time-complexity and 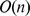 memory-complexity. One approach to reduce the memory complexity of DBSCAN would be to implement a grid-based density clustering algorithm that operates on cell densities rather than individual data points. Such algorithms exist [21]; however, employing a grid may compromise the resolution at which a misassembly can be identified. Finally, when coverage is high, subsampling the dataset can lower the memory load without sacrificing sensitivity.

Previous efforts have been made toward identification of misassemblies [22]. Implementations identify locations of putative misassemblies and require further manual inspection to remove these regions. The algorithm we developed for misassembly detection is conceptually similar to algorithms applied for segmental homology detection that operate by “chaining” homologous fragments into collinear blocks. Chaining algorithms such as FISH [23] and DAGChainer [24], are not permissive for this task, as they depend on a collinear arrangement of points. Because fragment lengths vary in size, points of mapped read pairs rarely fit this model of collinearity. The algorithm is also related to structural variant detection algorithms [25], [26]. Structural variant detection begins with mapping reads back to the reference and using read orientation information and mapping distance to identify anomalous pairs. In theory, some of these algorithms could also be employed to detect misassembly.

Our characterization of the performance of A5 also has limitations. We have only compared A5 to a single other assembler, SOAPdenovo, on a limited number of datasets. We chose this assembler because it is widely used and like A5 can assemble individual libraries without an additional mate pair library. Broad performance comparisons of many assemblers on many datasets is a major undertaking and we hope that A5 can be included in future comparisons like GAGE and the Assemblathon [14], [15].

## Design and Implementation

### A5 pipeline

**Figure 2:**
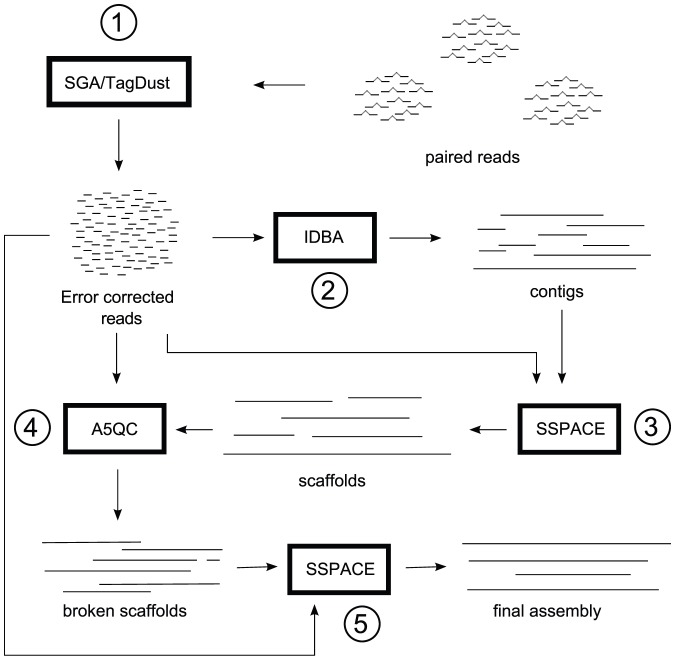
Overview of the stages in A5. The first stage of the A5 pipeline cleans reads, removing any contaminant reads and correcting base-call errors. Then the pipeline assembles contigs with IDBA using these error corrected reads. These contigs are then scaffolded using the original read set. Scaffolds are then checked for misassemblies, and broken at regions containing misassemblies. Finally, the broken scaffolds are rescaffolded using the original read set.

The A5 (**A**ndrew **A**nd **A**aron's **A**wesome **A**ssembly) pipeline consists of five stages: 1) read cleaning, 2) contigging, 3) scaffolding, 4) misassembly checking, and 5) rescaffolding. Figure 2 provides an overview of these stages.

#### Stage 1

For the first stage A5 uses two previously published programs. First, ambiguous and low quality portions of reads are removed from the dataset. Then sequencing errors are corrected in the reads. Both of these steps use tools from from the SGA software package [27]. Although many read error correction packages have been published, we found the implementation in SGA to have reasonable compute time and memory requirements compared to others while also providing good accuracy. Next, the pipeline applies Tagdust [5] to remove any sequencing adapter contamination that may be present in the data. The default set of adapter sequences used for screening include the standard Illumina TruSeq adapters and those used in Epicentre Nextera (transposon-catalyzed) library preparation protocols [17]. User-specified adapter sequences can be screened by adding them to a FastA file.

#### Stage 2

Using the newly cleaned reads derived from stage 1, stage 2 of the A5 pipeline builds contigs with the the assembler IDBA [28]. We selected IDBA due to its ability to produce long contigs in the presence of inconsistent depth of sequence coverage more robustly than other methods (data not shown). Like many current assembly algorithms, IDBA uses a *de Bruijn* graph-based algorithm to assemble contigs. A *de Bruijn* graph is a directed graph that represents overlap between all 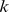-mers found in a nucleotide dataset. For a more complete description and comparison to other assembly approaches please see [29]. Many *de Bruijn*-based assemblers require the user to specify a single 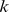-mer length, and the optimal choice of 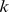 depends intimately on characteristics of the genome being assembled. Moreover it is possible that for a particular dataset with given read lengths and error profiles, different regions of the same genome may be optimally reconstructed by different values of 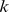. In contrast, IDBA simply requires a minimum and maximum value of 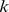 to use when processing the *de Bruijn* graph into contigs. This simplifies parameter choice. One final factor entering into the choice of IDBA was its ability to generate highly contiguous sequence even with unpaired sequence reads. Although assemblers using paired-end read information during contigging can often produce exceptional results [16], [30], we did not want to impose the requirement of paired reads (or multiple libraries with different insert sizes) upon users of the pipeline. This keeps applicability of A5 as broad as possible.

#### Stage 3

In stage 3 of A5, contigs are scaffolded and extended using the software SSPACE [9].

#### Stage 4

In stage 4 of A5, crude scaffolds are subjected to a quality control check for misassemblies. An undesirable side-effect of using a contigging algorithm that is unaware of read pairing information is that misassemblies can occur in contigs that could have been avoided if the longer-range linkage information present in read pairs (or long reads) had been used. As described in Results below, we observe occasional misassemblies in the contigs generated by IDBA. Although the version of IDBA currently incorporated into A5 (v0.20) has an option to use pairing information, it has little effect on the assembly (data not shown). Cleaned reads are mapped back to crude scaffolds using the read mapping software, BWA [3]. Custom code (described in detail below in section 0) is then used to extract all read pairs that are discordant with the crude scaffold assembly and two-dimensional spatial clustering [32] is used to identify clusters of discordant read pairs that are suggestive of a misassembly. The A5 pipeline then breaks the crude scaffolds at the estimated position of the misassembly.

#### Stage 5

Finally, in stage 5 the broken-up scaffolds are rescaffolded using SSPACE [9].

### Automated parameter selection

Most currently available assembly programs have a wide variety of parameters which must be specified by the user, and some of these can have a profound impact on the quality of the resulting assembly. The software employed within A5 is no exception. Often these parameters require dataset-specific tuning. A common approach employed by the hapless bioinformatician involves repeatedly executing the assembly software and evaluating the results until a perceived optimum has been achieved (or a pressing deadline looms). Scripts for automating this iterative tuning procedure have been developed [33], however, it is not always feasible, depending on available compute resources and the size of the dataset. The A5 pipeline avoids the problem for users by calculating reasonable parameters for each stage of the pipeline using values derived from the data itself. In some cases, default parameters have been set to data-independent values. Supplementary Text S1 summarizes the many parameters in the pipeline and Table S1 describes how they are set.

### Automated misassembly quality control

After crude scaffolds have been built, A5 performs an automated quality control step.

**Figure 3:**
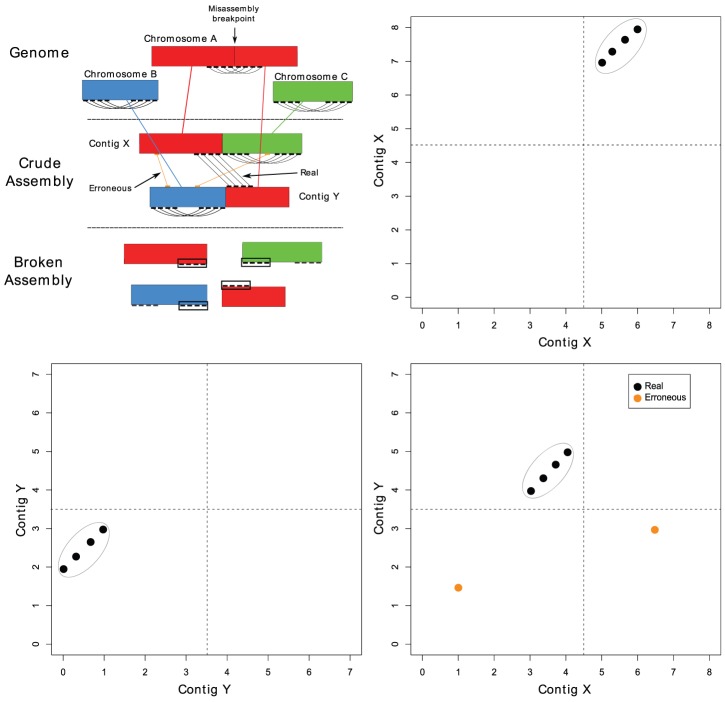
Demonstration of the automated misassembly quality control process. **Upper Left:** A hypothetical whole genome alignment of an assembly containing misassemblies relative to the true genome, consisting of three circular chromosomes, and the resulting broken assembly. Red, green, and blue lines connect aligned regions. Black connecting lines represent real paired read connections between contigs and orange connecting lines represent erroneous connections. Black boxes in the broken assembly highlight blocks identified by the DBSCAN algorithm. **Upper Right and Bottom Row:** Plots of connected points between contigs. Black and orange dots correspond to black and orange connections lines from left figure, respectively. Dotted lines correspond to misassembly breakpoints. Gray circles highlight the set of points that are clustered by DBSCAN.

As exemplified in Figure 3, reads are first mapped to scaffolds, and then read pairs are spatially clustered on the points where they map. After mapping, read pairs that support the current assembly architecture, which we refer to as *proper connections*, must be removed before spatial clustering. Without their removal, *proper connections* among read pairs would form large spatial clusters. Including these data in the clustering input would not only waste considerable computational resources but may also obscure or subsume clusters caused by local misassemblies in scaffolds.

Proper connections can be identified using the DNA fragment length (insert size) distribution of the library. However, two common features of Illumina datasets can skew the mean and inflate the variance estimates of the insert size distribution. The first of these features is referred to as a *shadow* library. Briefly, a shadow library is a population of small-insert (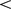
600 nt) paired end reads that are a product of imperfect construction of large-insert mate-pair libraries using the standard Illumina protocol. The Illumina mate-pair protocol involves circularization of fragments, further subfragmentation of the circular molecules, and purification of the linear subfragments containing the circularization junction. The purification of subfragments containing circularization junctions (from which the large-insert mate-pair reads derive) often fails to remove all DNA fragments lacking a circularization junction, those fragments yield the small insert read pairs termed a *shadow* library. The second feature that can interfere with insert size distribution calculations is inherent noise in the dataset. Such noise can be caused by chimeric fragments and ambiguous read mapping due to repetitive regions or highly erroneous reads.

### Accurate estimates of insert size distributions

To avoid including noise in mean and variance estimates from shadow libraries and other error sources, we perform a round of Expectation-Maximization (EM) clustering of insert sizes before calculating sample statistics [34]. Choice of the number of clusters 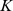 in the EM-clustering algorithm is derived from a preliminary estimation of the library insert size using the method implemented in BWA [31]. Libraries with a preliminary insert size estimate greater than 1000 bp are assumed to have been constructed using a mate-pair protocol, and therefore may contain a paired-end short insert shadow library in addition to the large insert mate-pair library. To separate the short insert library from the large insert library, 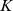 is set to 3: one cluster for improper connections, one cluster for the short insert shadow library, and one for the desired large insert library. If the preliminary insert size estimation is less than 1000 bp, the library is assumed to have been constructed using a paired-end protocol, and 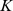 is set to 2: one for improper connections and one for the short-insert library. Clusters returned from EM-clustering are identified as containing improper connections if they have high variance, defined as 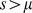, and proper connections if they have low variance (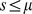), where 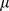 is the mean insert of pairs within the cluster, and 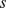 is the standard deviation. In practice, the 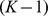
th lowest-variance clusters are identified as proper connections. Each low-variance cluster is then used to remove mapped read pairs having inserts in the range 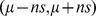, where 
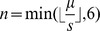
. The remaining read pairs represent improper connections and may contain clusters suggestive of misassembly.

After proper connections have been removed, misassemblies are identified by locating clusters of many read pairs mapped within a scaffold or between two scaffolds. We treat the mapped read pairs as *points* in two-dimensional spaces defined by each possible scaffold pair and self-pair. When the outer boundaries of a cluster of points is projected back onto the one dimensional sequence(s), we call the resulting intervals *blocks*. These blocks define regions of misassembly.

To identify blocks, we use the spatial clustering algorithm DBSCAN to cluster points in each of these 2-dimensional spaces [32]. The two key parameters of DBSCAN are 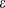, the maximum allowed distance between two points in a cluster and 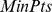, the minimum number of points allowed in a cluster. The first parameter is used to locate the neighboring points of each point, where a point 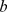 is considered a neighbor of point 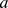 if 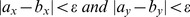. We set 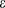 by modelling read mapping positions as a Bernoulli process. The probability of success 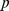 in the Bernoulli process is set by calculating a minimum read mapping frequency across the genome assembly. This is done by partitioning the assembly into windows of length 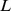, where 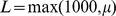 for a library with mean insert 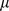. Let 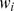 be the 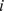
th window and 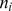 be the number of reads that map to 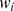. We then set 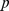 according to the following equation
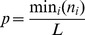
(1)The rationale for using the portion of the crude scaffold assembly with the fewest mapped reads is that in practice, sequencing coverage is often highly variable, with some regions receiving excessive coverage and others receiving little. This variation in coverage can be caused by systematic biases in the library construction and sequencing procedures, including fragmentation bias, PCR bias, and uneven representation of genomic DNA after DNA extraction. By estimating this parameter on a region of low coverage, we ensure sensitivity to detect misassemblies in low-coverage regions.

Assuming the positions of mapped reads follow a Bernoulli process, the distance between two independent reads in a sequence follows a geometric distribution with parameter 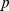. We derive a maximum allowable distance, 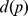, between two points (mapped reads) in one sequence by selecting the 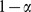 quantile of a geometric distribution with parameter 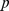. This is done by setting the cumulative distribution function, 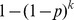, equal to 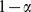 and solving for 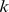:
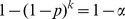


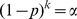


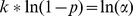


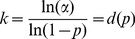
(2)for some 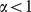. In practice we set 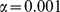, to select the 99.9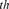 quantile. Furthermore, we assume overlapping reads belong to the same block, and set 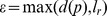 where 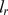 is the read length. The second parameter of the DBSCAN algorithm, 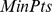, is set to be the expected number of points in the minimum allowed block length in a region of minimal coverage. Assuming a block will consist of 3 points at minimum and allowing a maximum distance between consecutive points to be 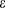, we allow the minimum block length to be 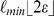. We then calculate the expected number of points in a window of length 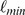 given that the probability of a read mapping to a single position is 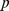, setting 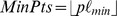.

Finally, regions of length 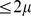 within individual scaffolds that are flanked by two blocks are identified as containing misassemblies and are removed from the assembly, breaking the scaffold into two subscaffolds. The removed region contains the misassembly breakpoint, but the exact position of the misassembly may not be well-defined in many cases, either due to lack of coverage by reads spanning that position or due to errors in the assembled sequence.

### Availability

Software for Linux and Mac OS X, along with source code is freely available from http://code.google.com/p/ngopt/ The source code has been licensed under the GNU Public License (GPL) v3.0.

## Supporting Information

Text S1 **Description internal assembly pipeline parameters.** The A5 pipeline incorporates many algorithms, each of which require certain parameters to be set. Each of these parameters is described in detail here.
(PDF)

Table S1
**Automatically set parameters.** Assembly parameters within the a5 pipeline and how their values are chosen. 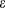 is the maximum inter-point distance used for spatial clustering and 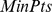 is the minimum number of points in a cluster.(PDF)

Table S2
**Assembly metrics for SOAPdenovo running on error corrected reads from** ***H. volcanii*** **DS2.** Reference-based assembly metrics for SOAPdenovo assemblies of *H. volcanii* DS2 (**Volc**) reads cleaned by stage 1 of the A5 pipeline. “scaf” indicates an assembly that has been scaffolded, while “ctg” indicates no scaffolding. Labels “-CDS”, “-N50”, and “-LCB” indicate SOAPdenovo assemblies run with parameter combinations that minimized broken coding sequences, maximized scaffold N50, and minimized LCB (Locally Collinear Block) count, respectively. SOAPdenovo with 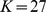 produced the best assemblies for -CDS, -N50, while 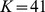 was optimal for -LCB. Contig statistics are on the contigs matching the optimal scaffold assemblies.(PDF)
