# Supplementary material for: An Integrated Pipeline for de Novo Assembly of Microbial Genomes
Source: PLoS One. 2012 Sep 13;7(9):e42304. doi: 10.1371/journal.pone.0042304 (PMC3441570; doi:10.1371/journal.pone.0042304)
Supplement: Text S1 — Description internal assembly pipeline parameters. The A5 pipeline incorporates many algorithms, each of which require certain parameters to be set. Each of these parameters is described in detail here. (PDF) [file pone.0042304.s001.pdf]

Text S1. Description of internal assembly pipeline parameters

The A5 pipeline incorporates many algorithms, each of which require certain parameters to be set. Supplementary Table S1 lists all of the parameters used in A5, along with how the value of that parameter is calculated. We now discuss the effect of each of these parameter settings in turn. The SGA Quality Trim parameter sets a PHRED Q-score cutoff [Ewing B, Green P (1998) Base-calling of automated sequencer traces using phred. II. Error probabilities. Genome Research 8: 186-194]. used for read trimming with the algorithm implemented in BWA [31]. The SGA Quality Filter parameter sets a maximum number of bases with a PHRED Q-score of below 3 that are permitted in a read before the read is discarded entirely. The SGA Min Read Length sets the minimum allowed read length after quality trimming, reads shorter than that will be discarded.

The IDBA min k-mer sets the starting k-mer size for *de Bruijn* graph construction, and the max k-mer sets the largest k that will be used during graph simplification.

The SSPACE Overhang MinOverlap sets the number of nucleotides that a read must overlap with an existing contig to be used for contig extension during scaffold gap filling. The SSPACE ExtendCall MinBases sets the minimum number of reads covering a base that are required to call the base during contig extension. The SSPACE minimum links sets the minimum number of read pairs that must be connecting a pair of contigs in order for them to be considered for scaffolding, the reads must map to the region of the contig expected based on the insert size distribution. For three contigs A, B, and C, where read pairs link A,B and A,C, the SSPACE Min Link Ratio sets the maximum allowable ratio between the number of links connecting A,C and A,B. If the ratio is below the threshold, A,B will be scaffolded, otherwise A will not be scaffolded. Here A,B and A,C have the highest and 2nd highest number of links between A and any other contig.

The SSPACE Merge MinOverlap sets the minimum number of bases that two scaffolded contigs must overlap in order to be merged into a single contig. The SSPACE Insert Mean is the mean value of the insert size for a paired-end or mate-pair library. The SSPACE Insert StDev is the standard deviation.
