# Supplementary material for: An Integrated Pipeline for de Novo Assembly of Microbial Genomes
Source: PLoS One. 2012 Sep 13;7(9):e42304. doi: 10.1371/journal.pone.0042304 (PMC3441570; doi:10.1371/journal.pone.0042304)
Supplement: Table S1 — Automatically set parameters. Assembly parameters within the a5 pipeline and how their values are chosen. is the maximum inter-point distance used for spatial clustering and is the minimum number of points in a cluster. (PDF) [file pone.0042304.s002.pdf]

Table S1. Automatically set parameters

Parameter	Stage	Default setting
SGA Quality Trim (-q)	1	fixed to 10, not optimized
SGA Quality Filter (-f)	1	fixed to 20, not optimized
SGA Min Read Length (-m)	1	fixed to 30, this should be larger than IDBA's min k-mer
IDBA min k-mer	2	29, most k-mers of this size are unique in a microbial genome but larger values may be bett[illegible]
IDBA max k-mer	2	maximum read length of trimmed reads
SSPACE Overhang MinOverlap (-m)	3,5	Max of 15 and floor of binary logarithm of genome size, plus 3.99
SSPACE ExtendCall MinBases (-o)	3,5	fixed to 1
SSPACE Minimum Links (-k)	3,5	base 1.4 logarithm of E_links, minus 11.5, where $E\_links = \frac{2c\mu}{l_r}$ and c is coverage
SSPACE Min Link Ratio (-a)	3,5	fixed to 0.6,0.3. These values give higher accuracy than SSPACE defaults
SSPACE Merge MinOverlap (-n)	3,5	floor of (binary logarithm of mean insert size, scaled by 1.24, plus 0.99)
SSPACE Insert Mean	3,5	Calculated using EM-clustering
SSPACE Insert StDev	3,5	Calculated using EM-clustering
DBSCAN ε	4	Derived from coverage as described in main text
DBSCAN $MinPts$	4	Derived from coverage as described in main text
