# Supplementary material for: An Integrated Pipeline for de Novo Assembly of Microbial Genomes
Source: PLoS One. 2012 Sep 13;7(9):e42304. doi: 10.1371/journal.pone.0042304 (PMC3441570; doi:10.1371/journal.pone.0042304)
Supplement: Table S2 — Assembly metrics for SOAPdenovo running on error corrected reads from H. volcanii DS2. Reference-based assembly metrics for SOAPdenovo assemblies of H. volcanii DS2 (Volc) reads cleaned by stage 1 of the A5 pipeline. “scaf” indicates an assembly that has been scaffolded, while “ctg” indicates no scaffolding. Labels “-CDS”, “-N50”, and “-LCB” indicate SOAPdenovo assemblies run with parameter combinations that minimized broken coding sequences, maximized scaffold N50, and minimized LCB (Locally Collinear Block) count, respectively. SOAPdenovo with produced the best assemblies for -CDS, -N50, while was optimal for -LCB. Contig statistics are on the contigs matching the optimal scaffold assemblies. (PDF) [file pone.0042304.s003.pdf]

Table S2. Assembly metrics for SOAPdenovo running on error corrected reads from *H. volcanii* DS2

	SOAPdenovo on clean reads					
Assembly	ctg-CDS	scaf-CDS	ctg-N50	scaf-N50	ctg-LCB	scaf-LCB
Sequence count	6965	237	6965	237	4101	227
N50	3781	125630	3781	125630	2457	85544
Miscalled bases	187	385	187	385	249	417
Uncalled bases	0	11567	0	11567	0	22162
Extra bases	31091	14830	31091	14830	65895	23369
Missing bases	154938	150235	154938	150235	160316	176787
Extra sequences	4964	92	4964	92	1206	81
Missing replicons	0	0	0	0	0	0
DCJ Distance	2005	150	2005	150	2897	148
LCB Count	9	13	9	13	10	10
Broken CDS	524	539	524	539	760	769
